# The β-Secretase 1 Enzyme as a Novel Therapeutic Target for Prostate Cancer

**DOI:** 10.3390/cancers16010010

**Published:** 2023-12-19

**Authors:** Hilal A. Rather, Sameh Almousa, Ashish Kumar, Mitu Sharma, Isabel Pennington, Susy Kim, Yixin Su, Yangen He, Abdollah R. Ghara, Kiran Kumar Solingapuram Sai, Nora M. Navone, Donald J. Vander Griend, Gagan Deep

**Affiliations:** 1 Department of Cancer Biology, Wake Forest School of Medicine, Winston-Salem, NC 27157, USA; hilal.rather@duke.edu (H.A.R.); salmousa@wakehealth.edu (S.A.); ashish.kumar@wakehealth.edu (A.K.); misharma@wakehealth.edu (M.S.); ipennington@wesleyan.edu (I.P.); sukim@wakehealth.edu (S.K.); ysu@wakehealth.edu (Y.S.); yahe@wakehealth.edu (Y.H.); aghara@wakehealth.edu (A.R.G.); 2 Department of Radiology, Wake Forest School of Medicine, Winston-Salem, NC 27157, USA; ksolinga@wakehealth.edu; 3 Atrium Health Wake Forest Baptist Comprehensive Cancer Center, Winston-Salem, NC 27157, USA; 4 Sticht Center for Healthy Aging and Alzheimer’s Prevention, Wake Forest School of Medicine, Winston-Salem, NC 27157, USA; 5 Department of Genitourinary Medical Oncology, David H. Koch Center for Applied Research of Genitourinary Cancers, The University of Texas MD Anderson Cancer Center, Houston, TX 77030, USA; nnavone@mdanderson.org; 6 Department of Pathology, The University of Illinois at Chicago, Chicago, IL 60612, USA; vandorama@gmail.com

**Keywords:** prostate cancer, BACE1, amyloid β peptide, MK-8931

## Abstract

**Simple Summary:**

Beta (β)-secretase 1, or β-site amyloid precursor protein-cleaving enzyme 1 (BACE1), is an enzyme involved in processing the amyloid precursor protein (APP) leading to generation of the amyloid β (Aβ) peptide. The role of this pathway is well-established in various neurological disorders, and in the present study, we report the role of the BACE1 enzyme in prostate cancer (PCa) growth and progression. The expression of BACE1 and its downstream product, i.e., Aβ1-42 was observed in various PCa tissue samples and BACE1 activity was confirmed across various PCa cell lines. BACE1 inhibitor (MK-8931) treatment reduced the PCa cell proliferation and its in vivo administration inhibited TRAMP-C1 allograft growth in immuno-competent C57BL/6 mice. Overall, this study suggests BACE1 as a novel therapeutic target in PCa.

**Abstract:**

Recent studies have demonstrated the association of APP and Aβ with cancer, suggesting that BACE1 may play an important role in carcinogenesis. In the present study, we assessed BACE1’s usefulness as a therapeutic target in prostate cancer (PCa). BACE1 expression was observed in human PCa tissue samples, patient-derived xenografts (PDX), human PCa xenograft tissue in nude mice, and transgenic adenocarcinoma of the mouse prostate (TRAMP) tissues by immunohistochemistry (IHC) analysis. Additionally, the downstream product of BACE1 activity, i.e., Aβ1-42 expression, was also observed in these PCa tissues by IHC as well as by PET imaging in TRAMP mice. Furthermore, BACE1 gene expression and activity was confirmed in several established PCa cell lines (LNCaP, C4-2B-enzalutamide sensitive [S], C4-2B-enzalutamide resistant [R], 22Rv1-S, 22Rv1-R, PC3, DU145, and TRAMP-C1) by real-time PCR and fluorometric assay, respectively. Treatment with a pharmacological inhibitor of BACE1 (MK-8931) strongly reduced the proliferation of PCa cells in in vitro and in vivo models, analyzed by multiple assays (MTT, clonogenic, and trypan blue exclusion assays and IHC). Cell cycle analyses revealed an increase in the sub-G1 population and a significant modulation in other cell cycle stages (G1/S/G2/M) following MK-8931 treatment. Most importantly, in vivo administration of MK-8931 intraperitoneal (30 mg/kg) strongly inhibited TRAMP-C1 allograft growth in immunocompetent C57BL/6 mice (approximately 81% decrease, *p* = 0.019). Furthermore, analysis of tumor tissue using the prostate cancer-specific pathway array revealed the alteration of several genes involved in PCa growth and progression including Forkhead O1 (FOXO1). All together, these findings suggest BACE1 as a novel therapeutic target in advanced PCa.

## 1. Introduction

It is estimated that about one in eight men will be diagnosed with prostate cancer (PCa) during their lifetime [1]. The 5-year survival rate for PCa patients is close to 100%, largely due to timely detection; still, 34,700 men are estimated to die due to PCa in 2023 [2]. The development of PCa is largely dependent on androgen and androgen receptor (AR) signaling; therefore, androgen-deprivation therapy (ADT) has been the mainstay of PCa treatment following surgery and radiotherapy. Though effective, several patients develop resistance to androgen deprivation, leading to castration-resistant PCa [3,4]. A subset of these tumor cells also undergoes a trans-differentiation process known as neuroendocrine differentiation [5]. Due to drug resistance and phenotypic changes acquired at later stages, the treatment options are limited, suggesting that additional measures are warranted to better understand and target this disease. 

Amyloid precursor protein (APP) is a transmembrane protein, which is sequentially cleaved by α-secretase or β-secretase (BACE1) followed by the γ-secretase complex to process into a non-amyloidogenic or amyloidogenic pathway. In the non-amyloidogenic processing, APP is cleaved by α-secretase followed by γ-secretase to produce the soluble amyloid precursor protein α (sAPPα), APP intracellular domain (AICD), and P3 fragment. In the amyloidogenic processing, BACE1 cleaves APP followed by γ-secretase to release soluble amyloid precursor protein β (sAPPβ), amyloid beta (Aβ), and AICD [6]. The role of APP and its processing secretases has been widely studied in neurodegenerative diseases [7,8,9]; however, few studies have also demonstrated their role in the development and progression of various cancers [10,11,12,13]. For example, APP was reported to promote cell proliferation in breast cancer by regulating p27kip1 [14] and promoting its migration and invasion by increasing the expression of different matrix metalloproteinases (MMPs) and activating the mitogen-activated protein kinase (MAPK) signaling pathway [15]. In PCa, APP was reported to regulate cell proliferation and migration, and its higher expression was correlated with poor prognosis [10,16]. Moreover, a higher expression of APP and its processing enzymes (β-secretase and γ-secretase) was reported in various PCa cells [13]. Several studies have also reported the role of BACE1 and Aβ in various cancers [11,12,17,18,19]. The inhibition of BACE1/2 using different inhibitors was shown to reduce the growth and viability of pancreatic cancer cells, suggesting that the cleavage products of APP and amyloid precursor-like protein 2 (APLP2) by BACE1/2 have an important role in cancer cells’ survival and proliferation [20]. Interestingly, BACE1 inhibition, with a selective chemical inhibitor MK-8931, reprogrammed the tumor-promoting macrophages into tumor-suppressive macrophages that promoted phagocytosis of glioma cells [11]. Recently, Aβ derived from cancer-associated fibroblasts, generated via the cleavage of APP by BACE1/2, was reported to drive neutrophil extracellular traps’ deposition in pancreatic cancer and melanoma [18]. In addition, in the xenograft model of inflammatory breast cancer, Aβ was found in extracellular aggregated forms [17]. Alongside that, melanoma cells require Aβ for brain metastasis. Aβ activated the anti-inflammatory phenotype of astrocytes and prevented the phagocytosis of melanoma via M2 polarization of microglia [12]. Despite studies suggesting the role of BACE1 and its product, Aβ, in various cancers, these molecular players have not been well-studied in PCa.

In the present study, we assessed the protein expression of BACE1 and/or Aβ in PCa tissues, allografts, ectopic, and patient-derived xenografts (PDX). The gene expression and activity of BACE1 were confirmed in various PCa cell lines. Lastly, the effect of BACE1 inhibition on PCa cell proliferation was assessed in cell culture and in vivo in an allograft model of PCa. Together, these results suggest BACE1 as a potential therapeutic target for PCa. 

## 2. Materials and Methods

### 2.1. Cell Culture 

Human non-malignant immortalized prostate epithelial cell line PWR-1E as well as human PCa cell lines LNCaP, PC3, DU145, and mouse TRAMP-C1 cancer cells were purchased from American Type Culture Collection (Manassas, VA, USA). PWR-1E cells were cultured in keratinocyte serum-free media with human recombinant epidermal growth factor and bovine pituitary extract (cat# 10724-011, Gibco, Thermo Fisher Scientific, Waltham, MA, USA). LNCaP cells were grown in RPMI 1640 medium (cat# 11875119, Gibco, Thermo Fisher Scientific, Waltham, MA, USA) with 10% fetal bovine serum (FBS) (cat# 16000044, Gibco, Thermo Fisher Scientific, Waltham, MA, USA). PC3 and DU145 cells were grown in RPMI 1640 media (cat# 11875119, Gibco, Thermo Fisher Scientific, Waltham, MA, USA) with 10% heat-inactivated FBS (cat# 10082147, Gibco, Thermo Fisher Scientific, Waltham, MA, USA). TRAMP-C1 cells were maintained in DMEM media supplemented with 5% FBS, 5% Nu-Serum (cat# CB55004, Fisher Scientific), 10 nM dehydroisoandrosterone (cat# 853-23-6, Millipore Sigma, Burlington, MA, USA), 0.005 mg/mL bovine insulin (cat# I6634, Millipore Sigma, Burlington, MA, USA), 1× GlutaMAX (cat# 35050061, Gibco, Thermo Fisher Scientific, Waltham, MA, USA) and 1× sodium pyruvate (cat# 11360070, Gibco, Thermo Fisher Scientific, Waltham, MA, USA). Enzalutamide-sensitive (S) and -resistant cells (C4-2B-S, C4-2B-R, 22Rv1-S, 22Rv1-R) were cultured in RPMI 1640 media with 10% FBS, and only resistant cells were cultured in 20 µM enzalutamide (cat# S1250, Selleckchem, Houston, TX, USA), as reported by us earlier [21]. A total of 100 U/mL penicillin G and 100 µg/mL streptomycin sulfate (cat# 15070063, Gibco, Thermo Fisher Scientific, Waltham, MA, USA) were added to each media. The cells were maintained in a humidified incubator with 5% CO_2_ at 37 °C. 

### 2.2. Animal Experiment

All mice were housed in accordance with the protocol approved by the Institutional Animal Care and Use Committee at Wake Forest University Health Sciences (Winston-Salem, NC, USA). TRAMP mice (C57BL/6-Tg(TRAMP)8247Ng/J) were purchased from Jackson Labs (Bar Harbor, ME, USA) at 12 weeks of age and were given ad libitum food and water on a 12 h light–dark cycle. Mice were sacrificed at 44 weeks of age. PC3-PTXR (paclitaxel-resistant) and 22Rv1 xenografts were reported previously by our lab [22,23]. MDA-PCa-118-B 7c (PDX118) and MDA-PCa-174-6-5a (PDX174) were obtained from the Tissue Biospecimen and Pathology Resource at MD Anderson Cancer Center (Houston, TX, USA) and maintained in CB17-SCID mice (Envigo, Indianapolis, IN, USA). 

C57BL/6J mice were purchased from Jackson Labs (Bar Harbor, Maine) at 5–6 weeks of age. For allograft generation, 3 × 10^6^ TRAMP-C1 cells in 50 µL PBS were mixed with 50 µL Matrigel matrix (cat# 354248, Corning, NY, USA) and injected subcutaneously and bilaterally into flanks. After one week, mice received intraperitoneal injections of either MK-8931 (30 mg/kg) or PBS five days a week for five weeks. Tumor growth was assessed with calipers, and volume (mm^3^) was calculated as width (mm)^2^ × length (mm) × 0.52. Mice were sacrificed at the end and tissues were collected. 

### 2.3. Archived Tissues and Immunohistochemistry (IHC)

Formalin-fixed paraffin-embedded human tissue array was procured from Tumor Tissue and Pathology Shared Resource of the Wake Forest Baptist Comprehensive Cancer Center. This array contained tissues samples from seminal vesicles (SVs), normal prostate (NP), and PCa (Gleason’s score 6 to 9). 

Other tumor tissues were fixed in 10% buffered formalin for 24 h and then stored in 70% ethanol until embedded in paraffin. The paraffin-embedded tissues were sectioned into 5 µm thick slices. For IHC, tissue sections were deparaffinized and rehydrated in xylene (Cat# X3P1GAL, Thermo Fisher Scientific, Waltham, MA, USA) and graded series of ethanol (Cat# A4124 Thermo Fisher Scientific, Waltham, MA, USA) with a final wash in distilled water. Antigen retrieval was performed using 10 mM citrate buffer (pH 6.0) at sub-boiling temperature for 1 h. Slides were washed in 0.2% Tween-PBS (PBST) before utilizing BLOXALL Blocking solution (cat# SP-6000-100, Vector Laboratories, Burlingame, CA, USA). Then, after washing in PBST, samples were incubated with 2.5% horse serum for 25 min. Slides were incubated overnight at 4 °C with either primary BACE1 antibody at 1:50 dilution (cat# 5606S, Cell Signaling Technologies, Danvers, MA, USA), Recombinant anti-Aβ1-42 antibody at 1:1000 dilution (cat# ab201060, Abcam, Waltham, MA, USA), or proliferating cell nuclear antigen (PCNA) antibody at 1:100 dilution (cat# 13-3900, Invitrogen, Carlsbad, CA, USA). The next day, slides were washed in PBST and incubated with ImmPRESS^®^-AP Horse Anti-Rabbit IgG Polymer Detection Kit, Alkaline Phosphatase (cat# MP-5401, Vector laboratories, Burlingame, CA, USA) or ImmPRESS^®^-AP Horse Anti-Mouse IgG Polymer Detection Kit, Alkaline Phosphatase (cat# MP-5402-15, Vector laboratories, Burlingame, CA, USA). For substrate development, we utilized ImmPACT Vector Red (cat# SK-5105, Vector laboratories, Burlingame, CA, USA), prepared according to the manufacturer’s instructions. Slides were washed twice in PBST and then counterstained with hematoxylin. Slides were submerged under running water to remove the extra dye. Slides were dehydrated in DI H2O, 70% ETOH, 95% ETOH, 100% ETOH, and xylene. Slides were mounted utilizing Cytoseal-60 mounting solution and covered with a coverslip. Slides were scanned by NanoZoomer (Hamamatsu, Japan) at 20×. IHC score (for BACE1) was based on the level of expression on a scale of 0 to 4 and the percentage of area expression in a visible field, where 0 denotes no expression and 4 denotes maximum expression. Finally, the percentage area expression was multiplied with the assigned 0-to-4 numbers to calculate the IHC score. The immunostaining for PCNA was evaluated by counting positive nuclei. The proportion of positive cells to the total number of cells in each field was calculated as a percentage. The scoring was conducted manually. 

### 2.4. Immunofluorescence (IF)

Formalin-fixed TRAMP tumor tissues were processed as mentioned above. After blocking, the samples were incubated overnight with BACE1 antibody at 1:50 dilution. The next day, samples were incubated with secondary anti-rabbit IgG Alexa Fluor^®^ 488 Conjugate (cat# 4412, Cell Signaling Technologies, Danvers, MA, USA) antibody for 2 h at room temperature. The slides were then washed with PBST and VECTASHIELD^®^ Antifade Mounting Medium with DAPI (cat# H-1200, Vector Laboratories, Burlingame, CA, USA) was used to mount slides. Images were captured using a confocal microscope (Olympus FV1200 Spectral Laser Scanning Confocal Microscope, Tokyo, Japan).

### 2.5. PET Imaging

Desmethyl-PiB was labeled with [^11^C]methyltriflate, produced from [^11^C]carbon dioxide, to obtain [^11^C]PiB [24]. [^11^C]PiB was formulated for injection in 10% ethanol in saline solution. The mean molar activity of [^11^C]PiB was 100 ± 27 GBq/μmol (mean ± SD) decay corrected to the end of synthesis. The radiochemical purity was > 95%. TRAMP mice were injected with [^11^C]PiB (~3.7 Mbq) 30 min before the PET scan. Mice were anesthetized with 2.5% isoflurane and kept warm over the heated bed. The eyes were protected from drying with ophthalmic gel (Oftagel, 25 mg/g; Santen Oy). All the microPET/CT scans were performed using TriFoil microPET/CT scanner. All the PET images were reconstructed and co-registered with the corresponding CT images and analyzed using the PMOD software (4.0 version). Tumor standard uptake values (SUVs) were calculated by manually drawing regions of interest (ROIs) on the tumor tissue from the fused PET-CT images.

### 2.6. Gene Expression 

RNA isolation was performed from PCa cells using RNeasy Mini kit (cat# 74106, Qiagen, Germantown, MD, USA) following the manufacturer’s instructions. cDNA synthesis was performed using 1 µg of RNA using High-Capacity cDNA Reverse Transcription Kit (cat# 368813 Applied Biosystems, Carlsbad, CA, USA). Quantitative polymeric chain reaction (qPCR) was performed using PowerUp™ SYBR™ Green PCR Master Mix (cat# 4A25742, Applied Biosystems, Carlsbad, CA, USA). mRNA expression was normalized with β-actin, and results are plotted as fold change (2^−ΔΔCt^).

### 2.7. Pathway Specific PCR Array

RNA was isolated after homogenizing the TRAMP-C1 tumor tissue in TRIzol (Thermo Fisher Scientific, Waltham, MA, USA) and utilizing RNeasy Mini kit (cat# 74106, Qiagen, Germantown, MD, USA). The cDNA was synthesized using 1 µg of total RNA in a 20 µL reaction. Mouse prostate cancer-specific pathway array was performed by RT2 profiler PCR array assay plate (Qiagen, Germantown, MD, USA) containing 84 genes and 5 loading controls, using the manufacturer’s recommendation. Altered gene expression is presented as a volcano plot. Differentially expressed genes with fold change (FC) log2(±0.06) and *p* value (≤0.05, or −log10 = (1.3)) are highlighted. 

### 2.8. BACE1 Activity Assay 

BACE1 activity assay kit (cat# ab282921, Abcam, Waltham, MA, USA) was utilized to perform the assay following the manufacturer’s instructions. Briefly, cells were trypsinized and 2 × 10^6^ cells were collected and lysed in 100 µL ice-cold BACE1 extraction buffer, incubated for 10 min, followed by centrifugation at 10,000× *g* for 5 min at 4 °C. The supernatant was collected and incubated with 4.32 M ammonium sulfate on ice for 30 min to precipitate proteins. The mixture was centrifuged at 10,000× *g* at 4 °C for 10 min. The supernatant was discarded, and the pellet was resuspended in 100 µL BACE1 assay buffer. 50 μL of samples were added to a 96-well black plate. A total of 50 µL of reaction mix containing 48 µL BACE1 assay buffer and 2 µL BACE1 substrate was added to each well and incubated for 10 min. The plate was measured immediately at Ex/Em = 345/500 nm at 37 °C. The BACE1 activity of samples was calculated using the following formula:

BACE1 Activity = B/(∆t × V) × D

where B is the EDANS amount from the standard curve (pmol), ∆t is the reaction time (min), V is the sample volume added into the reaction well (μL), and D is the dilution factor.

### 2.9. Colony Formation Assay

Single-cell suspensions were prepared by trypsinizing and resuspending cells in complete cell culture medium. A total of 200–1000 cells/well were plated in 6-well culture plates. The plates were incubated at 37 °C in a humidified atmosphere with 5% CO_2_ until colonies formed (6 to 8 days). After the incubation period, the colonies were fixed in 100% methanol, stained with crystal violet (cat# HT90132, Sigma-Aldrich, St. Louis, MO, USA), and counted (≥50 cells). 

### 2.10. MTT Assay

Cell proliferation as a function of metabolic activity was assessed utilizing 3-(4,5-dimethylthiazol-2-yl)-2,5-diphenyltetrazolium bromide (MTT) (cat# M5655-100MG, Sigma-Aldrich, St. Louis, MO, USA) assay. Cells were seeded in a 96-well plate. After 24 h, cells were either treated with DMSO (VC) or MK-8931 (verubecestat) (25 μM, 50 μM, 100 μM, and 200 μM) and incubated for desired time points. At the end of each time frame, 20 μL MTT solution (5 mg/mL) was added to each well and incubated for 2 h at 37 °C. After incubation, media were removed carefully, and then 200 μL DMSO was added to each well and incubated for 20 min at room temperature in the dark to dissolve formazan crystals. The absorbance was measured at 560 and 650 nm using Molecular Devices Precision microplate reader. (Molecular Devices, LLC, San Jose, CA, USA).

### 2.11. Trypan Blue Assay

A total of 5 × 10^4^ cells were seeded in 6-well plates. After 24 h, cells were treated with MK-8931 (25 μM, 50 μM and 100 μM). Then, after 24 h, 48 h, and 72 h treatments, cells were trypsinized and centrifuged at 2000× *g* for 5 min. After cells’ resuspension in PBS, 0.4% trypan blue solution (cat# 15250061, Gibco, Thermo Fisher Scientific, Waltham, MA, USA) was added, and the number of live and dead cells was counted using a hemocytometer. 

### 2.12. Cell Cycle Analysis

A total of 5 × 10^4^ cells were seeded in 6-well plates. After 24 h, cells were treated with MK-8931 (25 μM, 50 μM, and 100 μM). Then, after 72 h of treatment, cells were trypsinized and centrifuged. Cells were fixed overnight in cold ethanol at 4 °C. The next day, cells were washed twice with PBS and then treated with 100 µL of a 100 µg/mL stock of RNase (cat# 12091021, Invitrogen, Thermo Fisher, Waltham, MA, USA) for 30 min at room temperature. Cells were stained with 100 µL propidium iodide solution (75 µg/mL) (cat# P4864, Sigma-Aldrich, St. Louis, MO, USA) and analyzed by flow cytometer (CytoFlex, Beckman Coulter Life Science, Indianapolis, IN, USA) for cell cycle distribution analysis.

### 2.13. Statistical Analysis 

GraphPad Prism 9 software (GraphPad, San Diego, CA, USA) was used for all statistical analyses. All experiments were performed at least in triplicate for each set of experiments. Analysis was performed either using an unpaired *t*-test or ordinary one-way ANOVA, and results are plotted as mean ± SEM. *p*-value < 0.05 was considered as significant. 

## 3. Results

### 3.1. BACE1 Expression in PCa 

**Figure 1:**
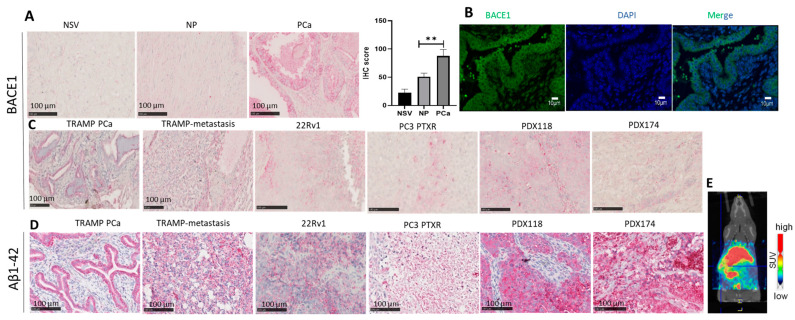
BACE1 and Aβ1-42 expression in PCa. (**A**) Representative IHC images showing the expression of BACE1 observed in normal seminal vesicles (NSVs) (*n* = 4), normal prostate tissue (NP), (*n* = 17) and localized PCa (*n* = 28) presented on a tissue array. The scale bar (100 µm) is shown on the images. Histogram representing IHC score for BACE1 expression calculated from the IHC images as mentioned in the methods. Analysis was performed using unpaired t-test with Welch’s correction and results are plotted as mean ± SEM, ** *p* < 0.001. (**B**) Representative immunofluorescence images showing the BACE1 expression (green) in TRAMP primary tumors (*n* = 3); DAPI was used as nuclear counterstain (blue). The scale bar (10 µm) is shown on the images. (**C**) Representative images of BACE1 expression in TRAMP primary tumors (*n* = 5), TRAMP abdominal cavity metastasis tumors (*n* = 3), 22Rv1 xenografts (*n* = 3), PC3-PTXR xenografts (*n* = 3), PDX118 xenografts (*n* = 3), and PDX174 (*n* = 3) xenografts. (**D**) Representative Aβ1-42 IHC images for TRAMP primary tumors (*n* = 5); TRAMP abdominal cavity metastasis tumors (*n* = 3); and 22Rv1 (*n* = 3), PC3-PTXR (*n* = 3), PDX118 (*n* = 3), and PDX174 (*n* = 3) xenografts. The scale bar (100 µm) is shown on the images. (**E**) Representative microPET/CT image of TRAMP PCa tumors 0–30 min after [^11^C]PiB injection in TRAMP mice (*n* = 2).

We first characterized the BACE1 expression in healthy and PCa tissues using human tissue arrays. The results revealed that BACE1 is expressed in normal human seminal vesicles (NSVs), surrounding normal prostate tissue (NP), and PCa tissue. However, the expression of BACE1 was relatively higher in human PCa tumor tissue compared to that in the NSVs or NP (Figure 1A). The TRAMP mouse model closely mirrors the pathogenesis of human PCa [25]. Therefore, BACE1 expression was analyzed in TRAMP tumors by immunofluorescence (IF). The results confirmed BACE1 expression in TRAMP tumors (Figure 1B). The results were further validated by IHC analysis of the TRAMP primary and metastatic tumors (Figure 1C). Furthermore, BACE1 expression was confirmed by IHC analysis in 22Rv1 xenografts, PC3-paclitaxel-resistant xenografts, and patient-derived xenografts (PDX118 and PDX174) in mice (Figure 1C).

As mentioned above, BACE1 is a principal protease required to produce Aβ from the amyloid precursor protein. Therefore, immunostaining of Aβ1-42 deposits was performed to confirm the functional activity of BACE1 in prostate tumors. Immunostaining of the samples confirmed the Aβ1-42 deposition in TRAMP primary tumors, TRAMP abdominal metastasis, 22Rv1 xenografts, PC3-paclitaxel-resistant (PTXR) xenografts, and patient-derived xenografts (Figure 1D). The PET tracer [^11^C]PiB specifically binds to fibrillar amyloid-beta plaques. Therefore, Aβ1-42 deposition was confirmed by the [^11^C]PiIB PET imaging (SUV = g/mL) of TRAMP tumors (Figure 1E). 

### 3.2. BACE1 Gene Expression and Activity in PCa Cells 

**Figure 2:**
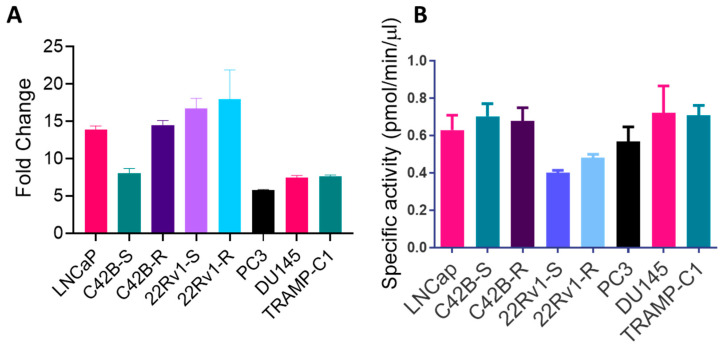
The gene expression and activity of BACE1 in multiple PCa cell lines. (**A**) The gene expression of BACE1 in various PCa cells was analyzed by RT-PCR; results are plotted as fold change compared to BACE1 expression in non-neoplastic PWR-1E cells. Each bar represents mean ± SEM (*n* = 3). (**B**) BACE1 activity of PCa cells. Each bar represents mean ± SEM (*n* = 3).

After confirming the expression of BACE1 in various PCa tumors, we aimed to investigate the expression and activity of BACE1 in various PCa cell lines. Therefore, we investigated the BACE1 mRNA expression in a broad range of human PCa cells (LNCaP, C42B-S, C42B-R, 22Rv1-S, 22Rv1-R, PC3, and DU145) and mouse PCa TRAMP-C1 cells. The results demonstrated a higher expression of BACE1 mRNA in all PCa cell lines compared to its expression in non-neoplastic PWR-1E cells (Figure 2A). The activity of BACE1 was also observed in all PCa cell lines utilizing a fluorescence-based BACE1 activity assay (Figure 2B). 

### 3.3. BACE1 Inhibition Causes Reduced Cell Viability in PCa Cells 

**Figure 3:**
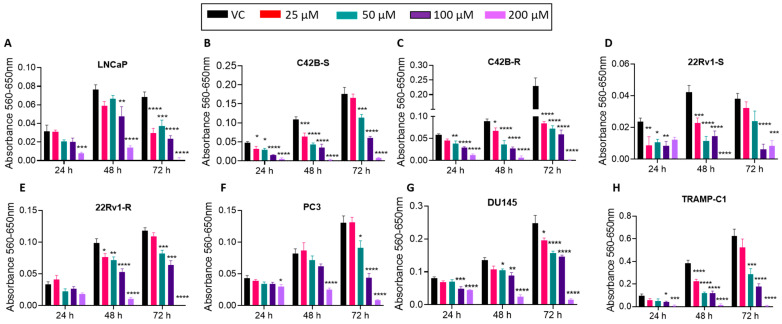
BACE1 inhibitor treatment reduces viability in multiple PCa cell lines. (**A**–**H**) The mentioned PCa cells (*n* = 6 replicates per group, repeated at least once) were treated with vehicle control (DMSO) or MK-8931 (25–200 μM) for 24–72 h, and cell viability was analyzed in MTT assay. Analysis was performed using one-way ANOVA, and results are plotted as mean ± SEM. * *p* < 0.05, ** *p* < 0.001, *** *p* < 0.001, and **** *p* < 0.0001.

**Figure 4:**
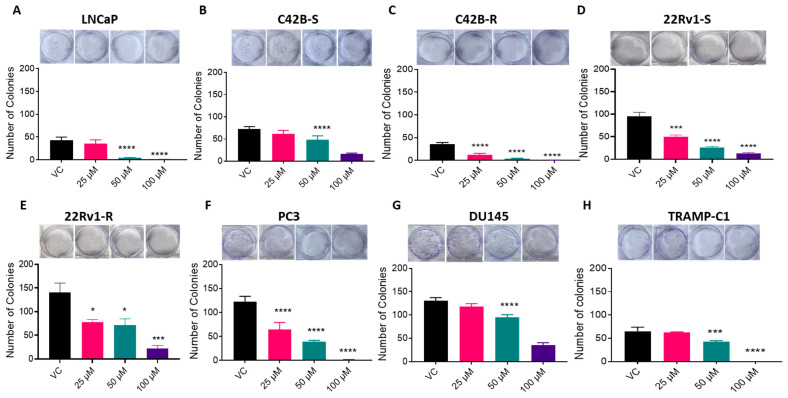
BACE1 inhibitor treatment reduces clonogenicity in multiple PCa cell lines. (**A**–**H**) The mentioned PCa cells (*n* = 3 per group, performed twice) were treated with vehicle control (DMSO) or MK-8931 (25–100 μM) for the defined duration, and the number of colonies (≥50 cells) was counted at the end. Analysis was performed by one-way ANOVA and presented as mean ± SEM. * *p* < 0.05, *** *p* < 0.001, and **** *p* < 0.0001.

To investigate the role of BACE1 in the viability of PCa cells, cells were treated with a potent and selective pharmacological inhibitor of BACE1 (MK-8931) [26], and an MTT assay was performed. The results demonstrated that the MK-8931 treatment (25–200 µM) significantly decreased the viability of all the PCa cell lines tested after 24 h, 48 h, and 72 h of treatment (Figure 3). Next, we assessed the long-term effect of BACE1 inhibition on the growth of PCa cells in a colony formation assay. As shown in Figure 4, MK-8931 treatment (25–100 µM) strongly reduced the clonogenicity of PCa cells in a dose-dependent manner (Figure 4). 

### 3.4. Effect of BACE1 Inhibition on Cell Growth, Death, and Cell Cycle in Enzalutamide-Sensitive and -Resistant PCa Cells

**Figure 5:**
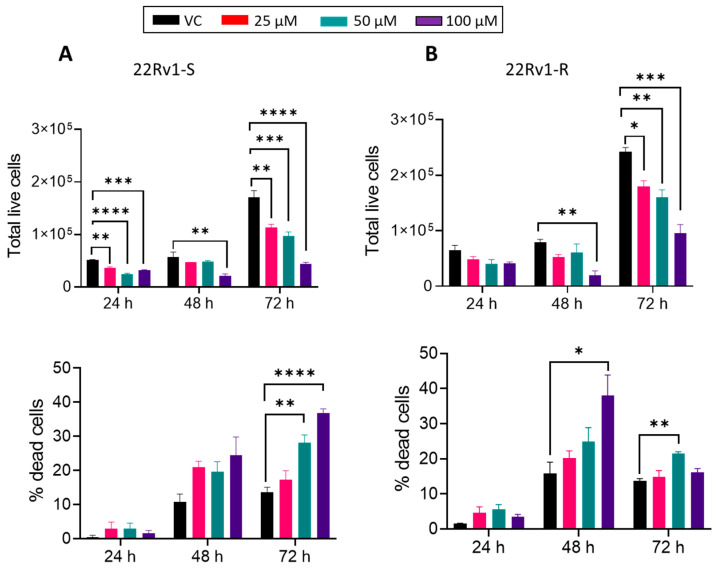
The effect of BACE1 inhibition on cell proliferation and death in enzalutamide-sensitive and -resistant 22Rv1 PCa cells. (**A**) 22Rv1-S and (**B**) 22Rv1-R cells were treated with vehicle control (DMSO) or MK-8931 (25–100 μM) for 24–72 h. At the end of each time point, total live cells and % dead cells were counted (*n* = 3/group). Analysis was performed using one-way ANOVA and results are plotted as mean ± SEM. * *p* < 0.05, ** *p* < 0.01, *** *p* < 0.001, and **** *p* < 0.0001.

Next, to discern the effect of BACE1 inhibition on cell proliferation and death on enzalutamide-sensitive and -resistant cells, we treated 22Rv1-S and 22Rv1-R cells with various doses of MK-8931 (25–100 µM) and performed the trypan blue exclusion assay. MK-8931 treatment reduced the live cell number and promoted cell death in both 22Rv1-S (Figure 5A) and 22Rv1-R cells (Figure 5B); however, the extent of cell death was more prominent at 72 h in 22Rv1-S cells and at 48 h in 22Rv1-R cells. 

**Figure 6:**
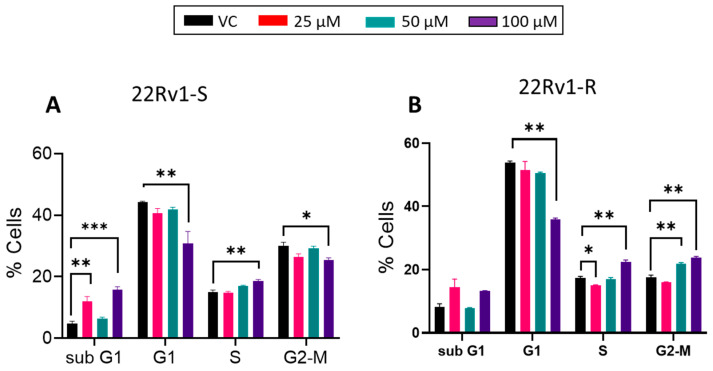
The effect of BACE1 inhibition on cell cycle distribution in enzalutamide-sensitive and -resistant 22Rv1 PCa cells. (**A**) 22Rv1-S and (**B**) 22Rv1-R cells were treated with vehicle control (DMSO) or MK-8931 (25–100 μM) for 72 h. At the end, cells (*n* = 3 replicates/group) were collected and analyzed for cell cycle distribution as described in the methods. Experiment was performed in triplicate (repeated once). Analysis was performed using one-way ANOVA, and results are plotted as mean ± SEM. * *p* < 0.05, ** *p* < 0.01 and *** *p* < 0.001.

Next, we characterized the cell cycle distribution in these cells following MK-8931 treatment. As shown in Figure 6, MK-8931 treatment increases the sub-G1 population in both 22Rv1-S and 22Rv1-R cells, though this increase was not dose-dependent. Furthermore, in 22Rv1-S, a significant decrease in the G1-phase cell population was observed at the 100 µM dose of MK-8931. In 22Rv1-R cells, MK-8931 treatment resulted in a decreased percentage of cells in the G1-phase, while a slight increase in S-phase and G2-M-phase cells was observed at the higher doses (50 and/or 100 µM). 

### 3.5. BACE1 Inhibitor Reduces the Growth of PCa Allografts in Mice without Any Toxicity

**Figure 7:**
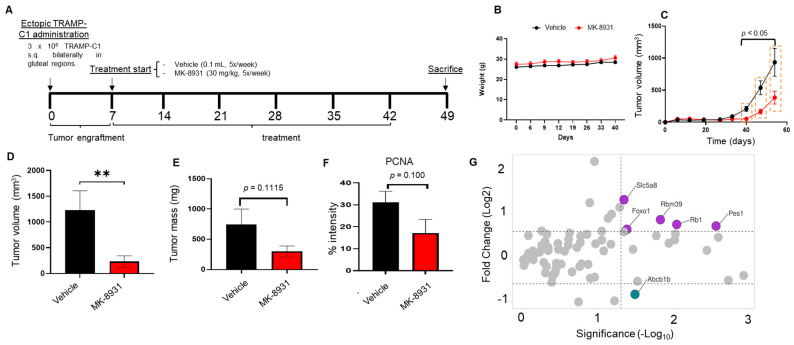
BACE1 inhibitor treatment reduces the in vivo growth of TRAMP-C1 allografts in syngeneic murine model. (**A**) Schematic representation of the experimental design. TRAMP-C1 cells were engrafted bilaterally into the flanks of wild-type C57BL6 mice. After one week of engraftment, mice were treated with either vehicle control or MK-8931 (30 mg/kg, IP) for 5 weeks. (**B**,**C**) Animal body weight and tumor volume was measured over the course of the study. Data are presented as mean ± SEM. Analysis was performed using multiple *t*-test analysis. Significant comparisons are outlined in dashed boxes for control versus MK-8931, *p* < 0.05. (**D**,**E**) Allografts were excised; volume (mm^3^) and weight (mg) were measured ex vivo. Data are presented as mean ± SEM. Analysis was performed using Student’s *t*-test. ** *p* = 0.019. (**F**) IHC analysis for PCNA expression in the tumor tissue of vehicle- and MK-8931 treated mice. Data are presented as mean ± SEM. Analysis was performed using Student’s *t*-test. (**G**) Pathway analysis of genes related to mouse prostate cancer pathway. Data are presented as volcano plot; significantly altered genes are highlighted as purple solid circles.

The effect of the pharmacologic inhibition of BACE1 on tumor growth was investigated using an in vivo syngeneic mouse PCa model. TRAMP-C1 cells were engrafted bilaterally into the flanks of wild-type C57BL6 mice, and a week later, mice were treated with either vehicle (control) or BACE1-inhibitor MK-8937 (30 mg/kg body weight, 5 times/week, i.p.). The experimental design is shown in Figure 7A. MK-8937 treatment did not show any toxic effects during the study duration, as reflected in the body weight of these mice (Figure 7B). Furthermore, mice treated with MK-8937 showed a significant reduction in tumor volume compared to that in the vehicle control group as early as 4 weeks post-treatment (Figure 7C). This was also reflected in significantly reduced tumor volumes in MK-8937 cohorts at sacrifice (approximately 81% decrease, *p* = 0.019) (Figure 7D). Interestingly, we found a decreasing trend in tumor weight (approximately 60%); however, there was no statistically significant difference between the two groups at the end of the study (*p* = 0.1115) (Figure 7E). The IHC analysis of tumor tissues for the expression of proliferation marker PCNA also showed a decreased trend in the MK-8937-treated mice, but this difference did not achieve statistical significance (*p* = 0.100) (Figure 7F). Furthermore, to identify the molecular pathways associated with the BACE1 inhibition, the prostate cancer-specific pathway array analysis was performed in tumor tissues of both vehicle- and MK-8937 treated mice. The expression of soluble carrier family 5 (iodide transporter), member 8 (Slc5a8), RNA binding motif protein 39 (Rbm39), retinoblastoma 1(Rb1), Pescadillo homolog 1 (Pes1), and Forkhead box O1 (FOXO1) was found to be significantly increased, while a reduced expression of ATP-binding cassette, sub-family B (MDR/TAP), and member 1A (Abcb1b) was observed in MK-8937-treated mice (Figure 7G).

## 4. Discussion

Despite the advancements in the diagnosis and treatment of PCa, several patients develop resistance to conventional therapeutic interventions due to the high heterogeneity in PCa, therapy-induced drug resistance, and cellular plasticity acquired due to reprogramming at the molecular and phenotypic levels [27]. These challenges demand the identification of new diagnostic and therapeutic targets for PCa. Studies have reported the effect of gonadal hormones on the Aβ peptide [28,29]. However, no previous study has yet reported the presence and role of Aβ in the PCa microenvironment as well as the expression of the key beta-secretase that regulates its production. Therefore, in the present study, we identified the role of BACE1 expression and Aβ1-42 deposits in PCa growth and progression. Our data suggest that BACE1 is widely expressed in PCa tumors with a higher expression compared to that in normal tissues. Importantly, we presented BACE1 activity in various PCa cells. [^11^C]PiB PET imaging results further validated BACE1-mediated production of Aβ1-42 deposition in TRAMP tumors. Most importantly, BACE1 inhibition resulted in PCa growth inhibition both in vitro and in vivo. 

There are only a few studies suggesting the molecular role of BACE1 in tumor progression. Although, Aβ produced by BACE1 could play a key role in the complex tumor microenvironment. For example, Kucheryavykh et al. reported that Aβ1-40 is present in gliomas and is either contributed by the cancer cells or has a systemic origin [30]. Kleffman et al. showed that Aβ activates the anti-inflammatory phenotype of astrocytes, suppresses neuro-inflammation, and prevents the phagocytosis of melanoma via the anti-inflammatory polarization of microglia to facilitate brain metastasis [12]. Furthermore, BACE1 reduced the phagocytosis of tumor cells in glioblastoma by activating the tumor-promoting macrophages via cleaving interleukin-6 receptor (IL-6R) and activating IL6/sIL-6R/STAT3 signaling [11]. We also observed reduced tumor growth with BACE1 inhibitor treatment in the TRAMP-C1 allografts in syngeneic mice, signifying the role of BACE1 in the growth of PCa. Furthermore, the BACE1 gene expression and enzymatic activity could indicate its functionality in PCa cells. The observed dose-dependent reduction in cell viability with MK-8931 treatment in the present study corroborates a previous study that demonstrated the decreased growth of pancreatic cells treated with β-secretase inhibitors. The treatment with β-secretase inhibitors was shown to prevent cleavage of amyloid precursor-like protein 2 to form C-terminal fragments, resulting in the decreased growth and viability of pancreatic cancer cells [20]. Furthermore, the Aβ1-42 oligomer is reported to activate the PI3K-Akt-mTOR pathway in neurons [31]. Activation of the PI3K-Akt-mTOR pathway plays a key role in cell viability, resistance against oxidative stress, tumor formation, disease progression, and therapeutic resistance in PCa [32,33,34,35]. Therefore, inhibition of Aβ1-42 formation by the BACE1 inhibitor could be related to the inhibition of downstream mitogenic and survival signaling pathways as a possible reason for the effect of MK-8931 on the viability of PCa cells. Although our study demonstrates the role of BACE1 in PCa, the involvement of the byproducts generated as a result of APP processing by BACE1 is not clear. For example, AICD, which is produced by BACE1 in association with γ secretase, could regulate the expression of various genes at transcriptional and translational levels [36,37]. Additionally, FOXO1, an important transcription factor regulating cell growth, differentiation, and metabolism, has also been shown to reduce the levels of Aβ by inhibiting pathways mediated by BACE1 [38] and also showed an inverse expression relation with BACE1 following treatment with the steroidal constituent of a plant extract, Physalin B [39]. We also observed an increased expression of FOXO1 with BACE1 inhibition, associated with reduced cell proliferation and reduced tumor volume. However, more studies are required to elucidate the mechanisms underlying the PCa growth inhibition by the BACE1 inhibitor. 

Our study signifies the use of pharmacological BACE1-inhibitor MK-8931 as a therapeutic drug against PCa. It has been extensively tested for its safety and toxicity in multiple species. The rats and monkeys treated chronically with MK-8931 showed no antemortem behavioral observation, clinical signs, or clinical chemistry changes attributable to neurodegeneration or hepatotoxicity, as well as no effects on serum glucose. In rabbits, hypopigmentation was observed starting about 2 to 3 weeks after the initiation of dosing, although without any sign of changes in skin architecture. We observed a similar hypopigmentation of the skin in MK-8931-treated mice. In humans, multiple doses of MK-8931 were generally well-tolerated in both healthy non-elderly adults and Alzheimer’s disease (AD) patients [26]. The X-ray co-crystal structure confirmed hydrogen-bonding interactions between the amidine moiety of MK-8931 and the BACE1 catalytic dyad. The diaryl amide substituent occupies the contiguous, relatively hydrophobic S1, S3, and S3sp sub-sites of BACE1, contributing to its high-affinity binding [26]. Importantly, this drug was developed for treating Alzheimer’s disease and other neurological disorders. Interestingly, one of the emerging side effects of androgen-deprivation therapy (ADT) in PCa patients is dementia. So, it needs to be studied whether ADT promotes BACE1 expression in the neurons, and, potentially, BACE1 inhibitor/s could be useful in preventing dementia development in PCa patients, along with reducing tumor burden. Interestingly, the results from the present study also showed efficacy against enzalutamide-resistant PCa cells, suggesting a broader applicability of BACE1 inhibitors in patients with advanced PCa.

## 5. Conclusions

In conclusion, the results of this study provided new insights into the role of BACE1 in the development and progression of PCa and highlight the potential of BACE1 inhibition as a therapeutic strategy. However, the in vivo effect of BACE1 inhibition was only explored in the subcutaneous allograft model of PCa; the effect further needs to be validated in orthotopic xenograft and transgenic models of PCa. The in vitro and in vivo effects were only studied with MK-8931 treatment, which inhibits BACE1 activity, but the study should also be validated with BACE1 knockdown/knockout and overexpressing cell lines and tumor models. Also, the detailed molecular mechanism and regulatory role of BACE1 and its downstream candidates for PCa growth and progression were not studied here. Therefore, more research is needed to fully understand the role of BACE1 and Aβ in PCa and to explore their potential as therapeutic targets for this disease. This study also suggests a potential distinct effect due to BACE1 inhibition on enzalutamide-sensitive and -resistant PCa cells, which also requires further investigation and validation. Lastly, in future studies, BACE1 inhibition could also be assessed in combination with immunotherapy as well as other treatment modalities. Overall, our findings about BACE1’s expression and role in PCa can provide a foundation for future research into the mechanisms by which BACE1 contributes to the development and progression of this disease.

## Data Availability

The data presented in this study can be shared on request.
